# Reversion of malignant phenotypes of human glioblastoma cells by *β*-elemene through *β*-catenin-mediated regulation of stemness-, differentiation- and epithelial-to-mesenchymal transition-related molecules

**DOI:** 10.1186/s12967-015-0727-2

**Published:** 2015-11-12

**Authors:** Tingzhun Zhu, Xiaoming Li, Lihan Luo, Xiaogang Wang, Zhiqing Li, Peng Xie, Xu Gao, Zhenquan Song, Jingyuan Su, Guobiao Liang

**Affiliations:** Department of Neurosurgery, General Hospital of Shenyang Military Area Command, No. 83, Wenhua Road, Shenhe District, Shenyang, 110840 China; Health Care Centre, Shenyang Entry-Exit Inspection and Quarantine Bureau, Shenyang, China

**Keywords:** *β*-Elemene, Glioblastoma, Malignant phenotype, *β*-Catenin

## Abstract

**Background:**

Glioblastoma is the most common and lethal type of primary brain tumor. *β*-Elemene, a natural plant drug extracted from *Curcuma wenyujin*, has shown strong anti-tumor effects in various tumors with low toxicity. However, the effects of *β*-elemene on malignant phenotypes of human glioblastoma cells remain to be elucidated. Here we evaluated the effects of *β*-elemene on cell proliferation, survival, stemness, differentiation and the epithelial-to-mesenchymal transition (EMT) in vitro and in vivo, and investigated the mechanisms underlying these effects.

**Methods:**

Human primary and U87 glioblastoma cells were treated with *β*-elemene, cell viability was measured using a cell counting kit-8 assay, and treated cells were evaluated by flow cytometry. Western blot analysis was carried out to determine the expression levels of stemness markers, differentiation-related molecules and EMT-related effectors. Transwell assays were performed to further determine EMT of glioblastoma cells. To evaluate the effect of *β*-elemene on glioblastoma in vivo, we subcutaneously injected glioblastoma cells into the flank of nude mice and then intraperitoneally injected NaCl or *β*-elemene. The tumor xenograft volumes were measured every 3 days and the expression of stemness-, differentiation- and EMT-related effectors was determined by Western blot assays in xenografts.

**Results:**

*β*-Elemene inhibited proliferation, promoted apoptosis, impaired invasiveness in glioblastoma cells and suppressed the growth of animal xenografts. The expression levels of the stemness markers CD133 and ATP-binding cassette subfamily G member 2 as well as the mesenchymal markers N-cadherin and *β*-catenin were significantly downregulated, whereas the expression levels of the differentiation-related effectors glial fibrillary acidic protein, Notch1, and sonic hedgehog as well as the epithelial marker E-cadherin were upregulated by *β*-elemene in vitro and in vivo. Interestingly, the expression of vimentin was increased by *β*-elemene in vitro; this result was opposite that for the in vivo procedure. Inhibiting *β*-catenin enhanced the anti-proliferative, EMT-inhibitory and specific marker expression-regulatory effects of *β*-elemene.

**Conclusions:**

*β*-Elemene reversed malignant phenotypes of human glioblastoma cells through *β*-catenin-involved regulation of stemness-, differentiation- and EMT-related molecules. *β*-Elemene represents a potentially valuable agent for glioblastoma therapy.

## Background

Glioblastoma is the most common and lethal type of primary brain tumor, accounting for approximately 52 % of primary intracranial tumors. Malignant phenotypes, including rapid proliferation, anti-apoptosis, resistance to radiation and chemotherapy and the tendency to peripherally invade, result in the reduction of therapeutic efficacy [1–4].

Elemene, an effective anti-tumor medicine, is extracted from *Curcuma wenyujin* as an essential oil mixture of *β*-, *γ*- and *δ*-elemene [5]. *β*-Elemene (PubChem CID: 10583), the major active anticancer component in the elemene mixture, displays high anti-proliferative activity and induces apoptosis in various tumors, such as glioma and breast carcinoma [6, 7]. We previously found that *β*-elemene arrested C6 and U87 glioblastoma cells in the G0/G1 phase of the cell cycle and inhibited cell proliferation by regulating the glia maturation factor *β*/mitogen-activated protein kinase kinase 3/6/p38 and extracellular signal-regulated kinase 1/2/B cell lymphoma 2/survivin pathways [7–10]. *β*-Elemene also inhibited cell proliferation and promoted differentiation of glioblastoma stem cells (GSCs) in vitro and in vivo [11]. However, the anti-glioblastoma effects of *β*-elemene and the mechanisms underlying these activities remain to be elucidated.

GSCs are a small population (1–10 %) of glioblastoma cells with some neural stem cells (NSCs) properties. GSCs are more difficult to kill than the differentiated population of glioblastoma cells owing to stronger phenotypes in promoting anti-apoptosis, invasion and resistance to chemoradiotherapy [11, 12]. Promoting GSC differentiation is a crucial therapeutic strategy in treating glioblastoma. Human CD133, a 120 kDa cell surface protein, has been accepted as the standard marker for the identification of GSCs, although CD133 may not be exclusively expressed on these cells or represent the ideal marker of this cell type [13–15]. ATP-binding cassette subfamily G member 2 (ABCG2), a chemotherapy resistance-related molecule, is strongly expressed in NSCs and tumor stem cells (TSCs) but is commonly inactive in further matured cells. Therefore, ABCG2 is considered to be an alternative GSC marker [16–20]. The expression of the differentiation marker glial fibrillary acidic protein (GFAP) is low in NSCs and GSCs and gradually increases during the cellular differentiation process. Additionally, Notch1 and Sonic hedgehog (SHH) are also closely associated with the differentiation of various tumor cells [21–23].

The epithelial-to-mesenchymal transition (EMT) is a unique invasion process that is typically accompanied by a decrease in the expression of the epithelial marker E-cadherin and an increase in the expression of the mesenchymal markers vimentin, N-cadherin and *β*-catenin. Inhibiting EMT would impair tumor cell invasiveness and improve patient prognosis [24, 25].

As the key regulator of both normal development and tumorigenesis, Wnt/*β*-catenin signaling plays a crucial role in the progression of human glioma. Accumulating evidence suggests that a complex crosstalk exists between the *β*-catenin pathway and various stemness-, differentiation- and EMT-related effectors [26–28].

In this study, we determined the effect of *β*-elemene on various malignant phenotypes in primary and U87 glioblastoma cells in vitro and in vivo.

## Methods

### Reagents and antibodies

*β*-Elemene (98 % purity) was obtained from Jingang Pharmaceutical Co. (Dalian, China). Antibodies against CD133, ABCG2 and GFAP that were used for Western blot and immunohistochemistry analyses were purchased from Boster Co., Ltd. (Wuhan, China). GAPDH antibody was obtained from Santa Cruz Biotechnology, Inc. (Santa Cruz, CA, USA), and the antibodies against Notch1, SHH, *β*-catenin, vimentin, E-cadherin and N-cadherin were purchased from Cell Signaling Technology, Inc. (Danvers, MA, USA). XAV939 (PubChem CID: 2726824) was purchased from Selleck Chemicals (Houston, TX, USA). Accutase Cell Dissociation Reagent was obtained from Invitrogen Corp. (Carlsbad, CA, USA). The cell counting kit-8 (CCK-8) was obtained from Dojindo Molecular Technologies, Inc. (Kumamoto, Japan). The Annexin *V*-FITC/propidium iodide (PI) apoptosis detection kit was purchased from BD Biosciences (Bedford, MA, USA). Nude mice were provided by the Experimental Animal Center of the Academy of Military Medical Sciences.

### Cell culture

The human U87 glioblastoma cell line was purchased from the Shanghai Cell Bank of the Chinese Academy of Sciences. The primary and U87 glioblastoma cells were maintained in Dulbecco’s modified eagle’s medium (DMEM; Hyclone Laboratories, Logan, UT, USA) supplemented with 10 % fetal calf serum (Invitrogen), 50 IU/ml penicillin (Invitrogen) and 50 mg/ml streptomycin (Invitrogen) and cultured at 37 °C in a humidified atmosphere containing 5 % CO_2_.

### Tumor specimens and primary cell cultures

**Fig. 1:**
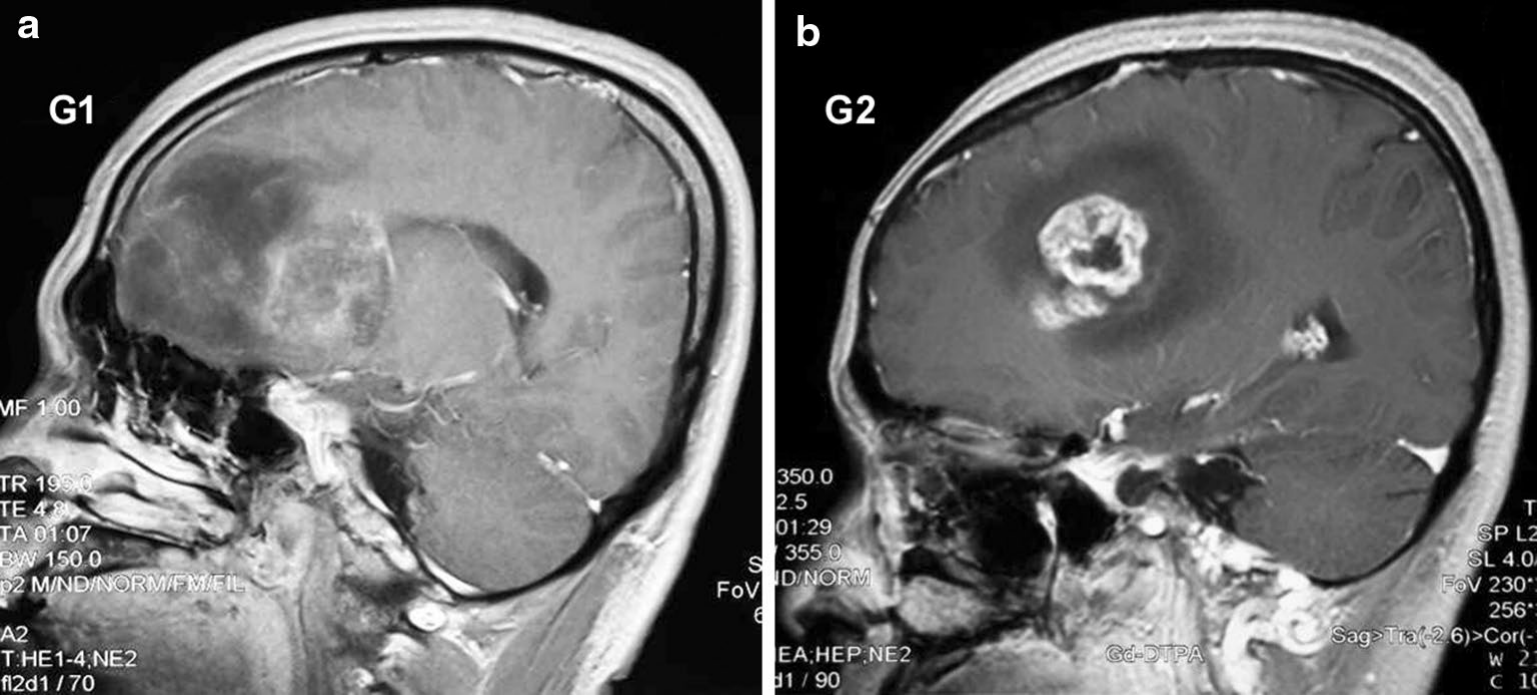
Head-enhanced MRI images for patients G1 and G2. **a** The head-enhanced MRI image of patient G1, who exhibited a glioblastoma in the *right* frontal lobe. **b** The head-enhanced MRI image of patient G2, who exhibited a glioblastoma in the *left* frontal lobe

Brain tumors located in different regions of the brain often show different symptoms, prognosis and even biological behaviors. To enhance the compatibility of our results, this study included two cases (G1: male, 46 years old, WHO grade III; G2: male, 39 years old, WHO grade IV) with glioblastoma in frontal lobes who received surgical resection of glioblastoma in the Department of Neurosurgery of the General Hospital of Shenyang Military Area Command. Each patient’s head-enhanced magnetic resonance imaging (MRI) images are shown in Fig. 1. This study was approved by the Ethics Committee of the General Hospital of Shenyang Military Area Command and abided by the Declaration of Helsinki; informed consent was obtained from all study participants. The G1 and G2 tumor samples were stored in sterile serum-free DMEM and processed within 0.5 h after resection. The tissues were cut into 1 mm^2^ pieces, washed with phosphate-buffered saline (PBS) and digested using 0.25 % trypsin at 37 °C for 15 min. The G1 and G2 primary glioblastoma cells were cultured in serum-containing DMEM after filtering through a 70 µm strainer (BD Biosciences).

### Immunohistochemistry

Paraffin sections of the glioblastoma tissues were obtained and deparaffinized. Antigen retrieval was performed using citrate buffer, pH 6.0 (Invitrogen). Non-specific sites were blocked by incubating the sections in 5 % bovine serum albumin (BSA) in a humidified chamber for 1 h at room temperature. The samples were incubated in 0.3 % H_2_O_2_ for 15 min to block endogenous peroxidase activity and then labeled with different primary antibodies (1:100 for CD133, ABCG2 and GFAP) for 1 h at room temperature. We used the Vectastain ABC kit (Vector Labs., Burlingame, CA, USA) and diaminobenzidine (Changdao Biotech, Shanghai, China) as a chromogen. Nuclear counterstaining of the sections was performed using hematoxylin. In all tissues, one section was stained without the primary antibody in parallel as a negative control.

### CCK-8 assay

Cell viability was evaluated using the CCK-8 assay in cells cultured in a 96-well plate in the exponential growth phase. Trypan blue staining confirmed >80 % cell viability, and the cells were treated according to the study design. Then, 10 µl of CCK-8 was added to each well and the mixture was incubated for 4 h at 37 °C. The optical density of each well was measured at 450 nm using a spectrophotometric microplate reader (Bio-Tek Instruments Inc., Winooski, VT, USA). Five replicate wells were used for each condition.

### Cell proliferation assay

Cells (4 × 10^5^ cells per well) were grown in six-well plates overnight and then treated with various concentrations of *β*-elemene for 24 h. Trypan blue staining confirmed >80 % cell viability, and cell numbers were determined by cell counting (Beckman Coulter, Miami, FL, USA).

### Detection of cell death using flow cytometry

Primary and U87 glioblastoma cells (6 × 10^5^ cells) were seeded on 6 cm diameter culture dishes, incubated for 24 h, and then treated with *β*-elemene at various concentrations for 24 h. Both live and dead cells (including both the adherent cells and the cells suspended in the medium) were collected, washed with PBS, and then resuspended in binding buffer (BD Biosciences), to which Annexin V-FITC and PI were added. Flow cytometry assay was performed to evaluate apoptosis. All in vitro experiments were conducted in triplicate.

### Western blot

Cells were lysed using RIPA buffer [50 mM Tris–HCl (pH 7.4), 1.0 % NP-40, 0.25 % Na-deoxycholate, 1 mM EDTA, 150 mM NaCl, 1 mM aprotinin, 1 mg/ml PMSF, 1 µg/ml pepstatin and 1 µg/ml leupeptin]. The total protein concentrations in the cellular extracts were measured using the BCA assay kit from Keygen Biotech. Co., Ltd. (Nanjing, China). After separation via 10 % sodium dodecyl sulfate–polyacrylamide gel electrophoresis, the proteins were transferred to nitrocellulose filter membranes (Bio-Rad, Hercules, CA, USA). The membranes were blocked using 5 % BSA in Tris-buffered saline containing Tween 20 at 4 °C overnight. The membranes were probed using various primary antibodies at 4 °C overnight, followed by incubation in horseradish peroxidase-conjugated secondary antibodies at 37 °C for 1.5 h. The membranes were exposed to an ECL system (Amersham Biosciences, Uppsala, Sweden), and chemiluminescence was detected by exposing the membranes to x-ray film (Fujifilm Co., Ltd., Tokyo, Japan). The results were digitized using Image Quant 5.2 software (Amersham), and the gray values of the bands were semi-quantitatively evaluated using Gel-Pro Analyzer 4.0 software (Media Cybernetics, Rockville, MD, USA). The gray values were normalized to those of GAPDH.

### In vitro invasion assays

Cell invasion assays were performed in 24-well plates equipped with 8 mm pore size chamber inserts (Corning, New York, NY, USA). Cells were diluted in serum-free culture medium and placed in upper wells (1 × 10^5^ cells per well) that were previously coated with Matrigel (BD Biosciences). Cells were suspended in 200 µl of serum-free DMEM (supplemented with *β*-elemene, dimethyl sulfoxide (DMSO) or 10 µM XAV939) upon seeding on the upper chamber. In the lower chamber, 500 µl of DMEM supplemented with 20 % fetal bovine serum was added. After incubation for 12 h at 37 °C, the membrane inserts were removed from the plate and the non-invading cells were removed from the upper surface of the membrane. The cells that moved to the bottom surface of the upper chamber were fixed using 100 % methanol for 15 min and stained with 0.1 % crystal violet for 30 min. Cells were imaged and counted in 16 fields using an inverted microscope (ECLIPSE TE2000-U, Nikon, Tokyo, Japan). The assays were conducted in triplicate.

### Transplantation of glioblastoma cells into nude mice and treatment of the animals

A total of 1 × 10^5^ glioblastoma cells (suspended in 0.2 ml DMEM) were subcutaneously injected into the right shoulder region of each 4-week-old female nude mouse. Various drugs were intraperitoneally injected beginning on day 6 after transplantation. The tumor volumes were measured every 3 days and were calculated according to the equation V = 1/2 × largest diameter × smallest diameter^2^ [8]. The tumors were weighed on day 21 after transplantation. All experimental procedures involving animals were also approved by the Institutional Animal Care and Use Committee of the General Hospital of Shenyang Military Area Command. All efforts were made to minimize animal suffering and discomfort and to reduce the number of animals used.

### Statistical analysis

The values were reported as the mean ± standard deviation (SD) of at least three independent experiments. The data were analyzed using Student’s t test for comparisons of two groups or using a one-way analysis of variance followed by Tukey’s post hoc test for multiple comparisons. Statistical significance was accepted at the level of *p* < 0.05 between different groups, and *p* < 0.01 was considered to be highly significant. The statistical analyses were performed using SPSS software version 16.0 (SPSS, Inc., Chicago, IL, USA).

## Results

### *β*-Elemene inhibited the proliferation of primary and U87 glioblastoma cells

**Fig. 2:**
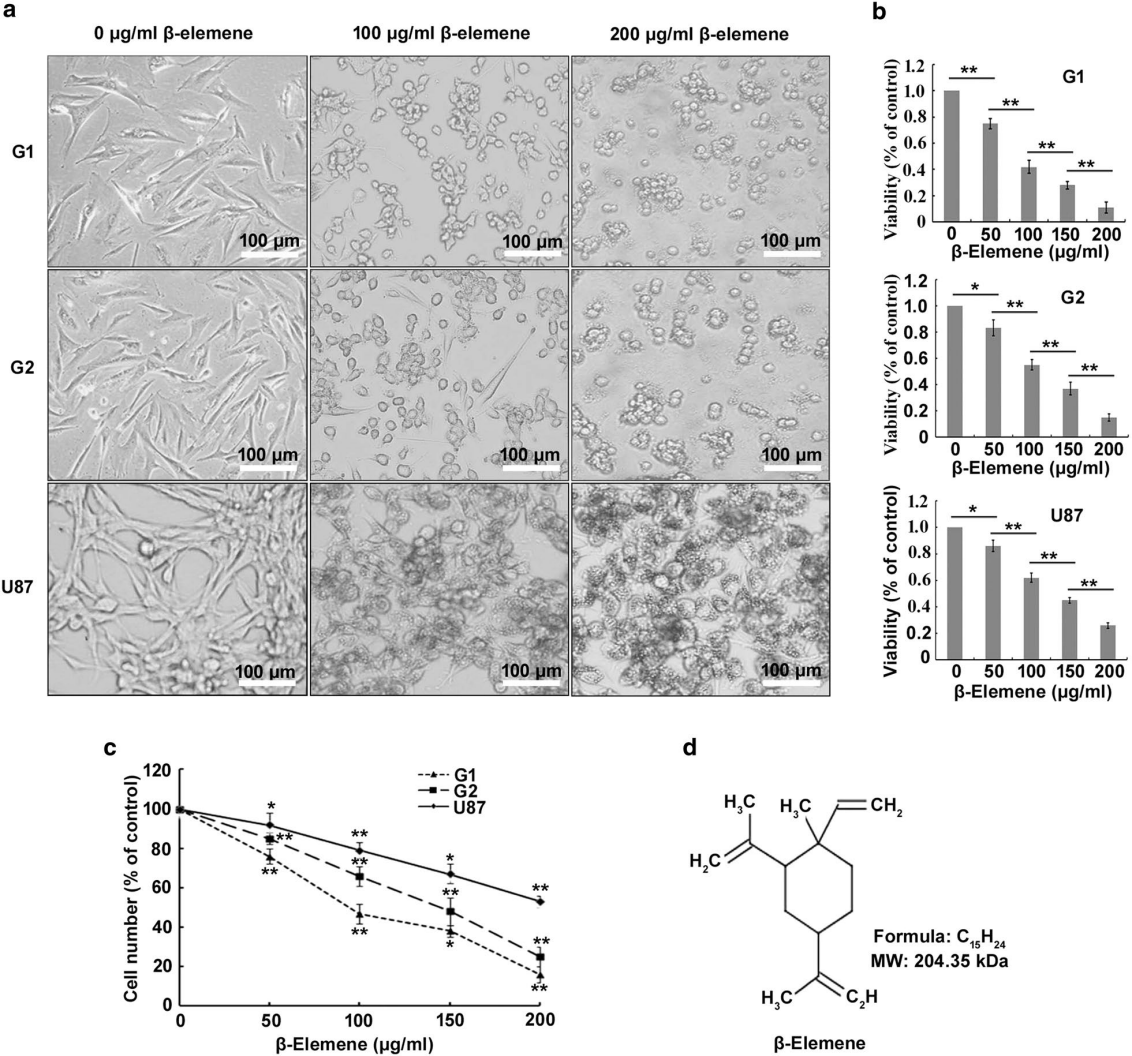
*β*-Elemene inhibited the proliferation and viability of glioblastoma cells. **a** G1 and G2 cells were fusiform or polygonal, displaying clear cellular atypia and pathological nuclear fission. Upon treatment with *β*-elemene, the glioblastoma cells gradually shrank, and a small proportion of the cells died and floated in the medium. **b** Cell viability was clearly inhibited by *β*-elemene in a concentration-dependent manner. **c** Cell numbers were impaired by *β*-elemene in a concentration-dependent manner. **d** The molecular structure of *β*-elemene. The values are presented as the mean ± SD (*p < 0.05, **p < 0.01). *Scale bar *  100 µm

To evaluate the anti-tumor effect of *β*-elemene on glioblastoma cells, G1, G2 and U87 cells were treated with *β*-elemene at 0 (untreated), 50, 100, 150 or 200 µg/ml for 24 h. G1 and G2 cells were fusiform or polygonal, displaying clear cellular atypia and pathological nuclear fission. Multinucleated or giant tumor cells were occasionally detected among the G1 and G2 cells. G1, G2 and U87 cells treated with *β*-elemene gradually shrank and a small proportion of the cells died and floated in the medium (Fig. 2a). CCK-8 assays showed that both viability and number of cells treated with *β*-elemene decreased in a dose-dependent manner (Fig. 2b, c). These results indicated that *β*-elemene inhibited the proliferation of human glioblastoma cells.

### *β*-Elemene induced glioblastoma cell apoptosis

**Fig. 3:**
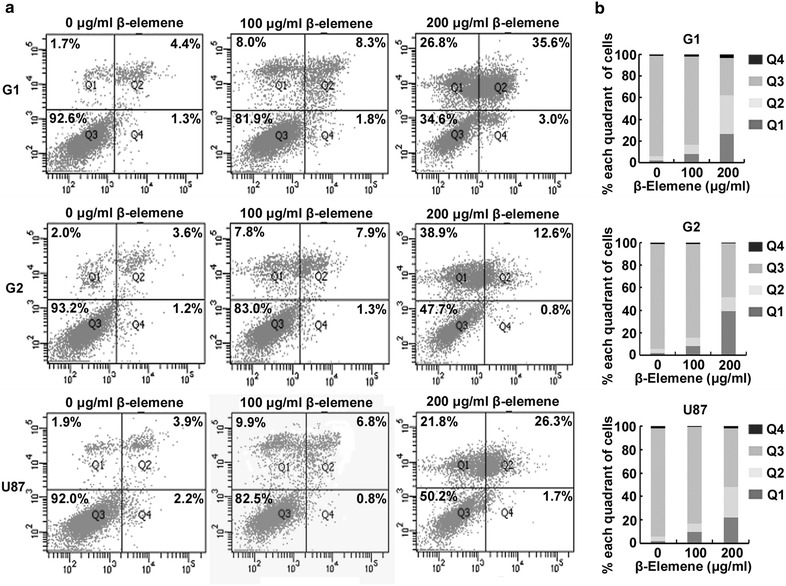
Induction of apoptosis in glioblastoma cells after incubation in *β*-elemene for 24 h. **a** Glioblastoma cells were treated with *β*-elemene for 24 h. Flow cytometry was performed using an Annexin V/PI detection kit. Upon treatment with *β*-elemene, the proportions of apoptotic cells clearly increased in a dose-dependent manner. Viable cells are located in the *lower left* quadrant, cells in the early apoptosis stage are located in the *lower right* quadrant, cells in the late apoptosis stage or already dead are located in the *top right* quadrant and necrotic cells are located in the *top left* quadrant. **b** The results in (**a**) are illustrated graphically. These results are representative of three independent experiments

To evaluate the effect of *β*-elemene on glioblastoma cell apoptosis, G1, G2 and U87 cells were treated with *β*-elemene at 0 (untreated), 100 or 200 µg/ml for 24 h. Both live and dead cells (including both the adherent cells and the cells suspended in the medium) were collected and flow cytometry was performed using an Annexin V/PI detection kit. More than 90 % of the untreated G1, G2 and U87 cells were viable. Figure 3 shows flow cytometry data from treated cells; viable cells are located in the lower left quadrant, cells in early apoptosis are located in the lower right quadrant, cells in late apoptosis stage or that are already dead are located in the top right quadrant and necrotic cells are located in the top left quadrant [29]. As the *β*-elemene dose increased, the proportions of apoptotic cells were dramatically increased. These results indicated that *β*-elemene induced glioblastoma cell apoptosis.

### *β*-Elemene regulation of the expression of stemness- and differentiation-related effectors in glioblastoma cells

**Fig. 4:**
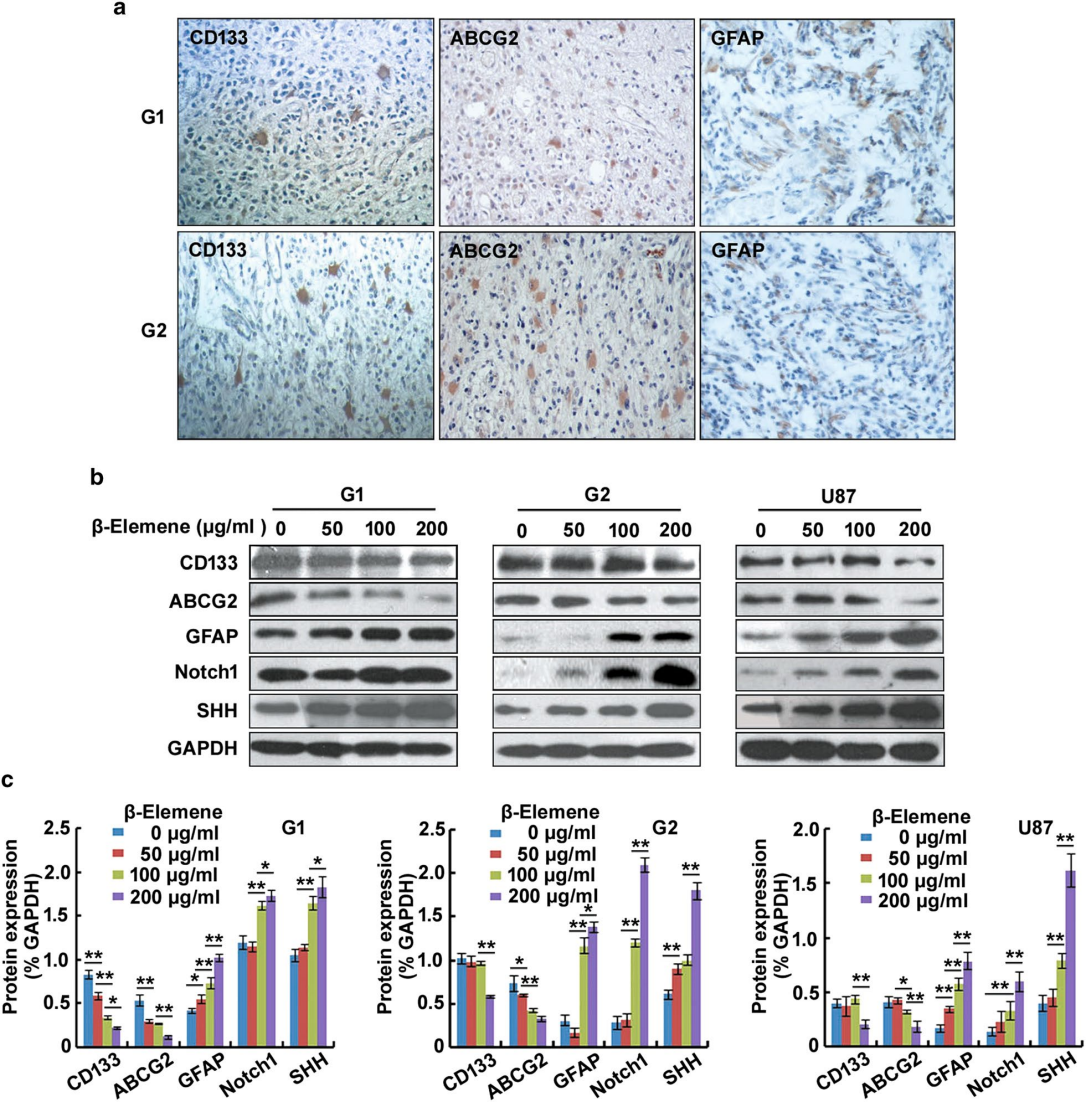
*β*-Elemene regulated the expression of stemness markers and differentiation-related effectors in glioblastoma cells. **a** CD133^+^ and ABCG2^+^ cells were sparsely distributed throughout both G1 and G2 tissues, and both CD133 and ABCG2 were localized to both the cytoplasm and cytomembrane. The expression of GFAP was higher in the G1 tissue than in the G2 tissue. **b** *β*-Elemene decreased the expression levels of CD133 and ABCG2 and increased the expression levels of GFAP, Notch1 and SHH in a dose-dependent manner. **c** The results of (**b**) were semi-quantitatively estimated using Gel-Pro Analyzer 4.0 software and are illustrated graphically. The results are representative of three independent experiments, and the values are presented as the mean ± SD (*p < 0.05, **p < 0.01)

We next performed immunohistochemical analysis to investigate the expression levels of stemness (CD133, ABCG2) and differentiation-related markers (GFAP) in human glioblastoma tissues. Both CD133- and ABCG2-positive cells were sparsely distributed throughout the glioblastoma tissues and both CD133 and ABCG2 were localized to both the cytoplasm and cytomembrane. The expression of GFAP was detected in both the G1 and G2 tissues and was higher in the G1 tissue than in the G2 tissue (Fig. 4a).

We next performed Western blot analysis to evaluate the expression levels of the stemness markers CD133 and ABCG2 and differentiation-related molecules GFAP, Notch2 and SHH in G1, G2 and U87 cells treated with 0, 50, 100 or 200 µg/ml *β*-elemene for 24 h. The expression levels of CD133 and ABCG2 were significantly downregulated by *β*-elemene whereas the expression levels of GFAP, Notch1 and SHH were upregulated in a dose-dependent manner (Fig. 4b, c). Together these results suggested that *β*-elemene inhibited the expression of stemness markers and increased the expression of differentiation-related effectors in glioblastoma cells in vitro.

### *β*-Elemene regulation of the expression of EMT-related effectors in glioblastoma cells in vitro

**Fig. 5:**
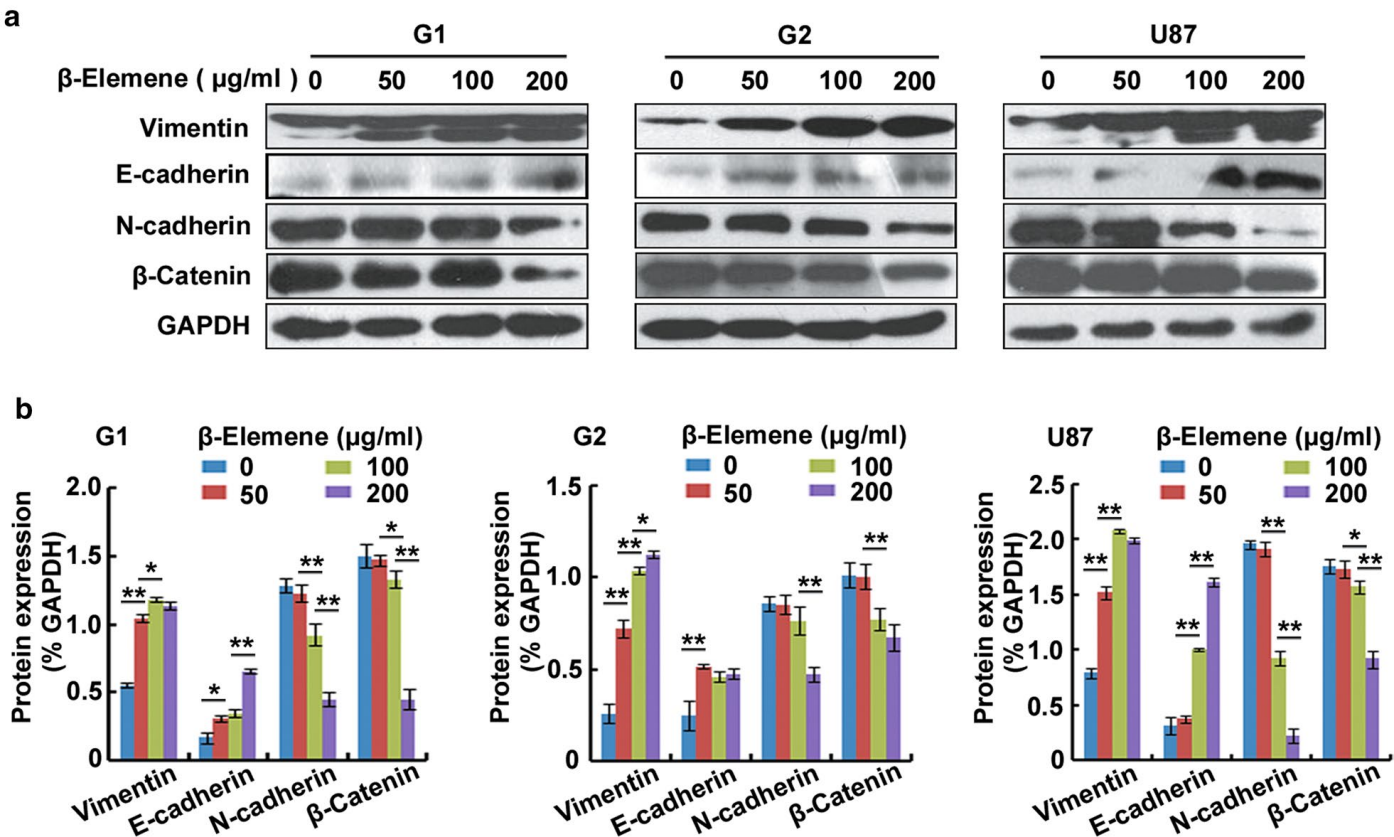
*β*-Elemene effect on the expression of EMT-related effectors in glioblastoma cells. Cells were treated with *β*-elemene at various doses for 24 h and analyzed by Western blot. **a** Compared with the untreated cells, the expression levels of vimentin and E-cadherin were significantly increased whereas the expression levels of N-cadherin and *β*-catenin were decreased in *β*-elemene-treated cells in a dose-dependent manner. **b** The results of (**a**) were semi-quantitatively estimated using Gel-Pro Analyzer 4.0 software and are illustrated graphically. The results are representative of three independent experiments, and the values are presented as the mean ± SD (*p < 0.05, **p < 0.01)

To evaluate the effect of *β*-elemene on the expression of EMT-related effectors, G1, G2 and U87 cells were treated with 0, 50, 100 or 200 µg/ml *β*-elemene for 24 h and Western blot analysis was performed to evaluate vimentin, E-cadherin, N-cadherin and *β*-catenin expression. The results revealed that *β*-elemene increased the expression levels of vimentin and E-cadherin and decreased the expression levels of N-cadherin and *β*-catenin (Fig. 5).

### *β*-Elemene decreased the invasiveness of glioblastoma cells by suppressing *β*-catenin expression

**Fig. 6:**
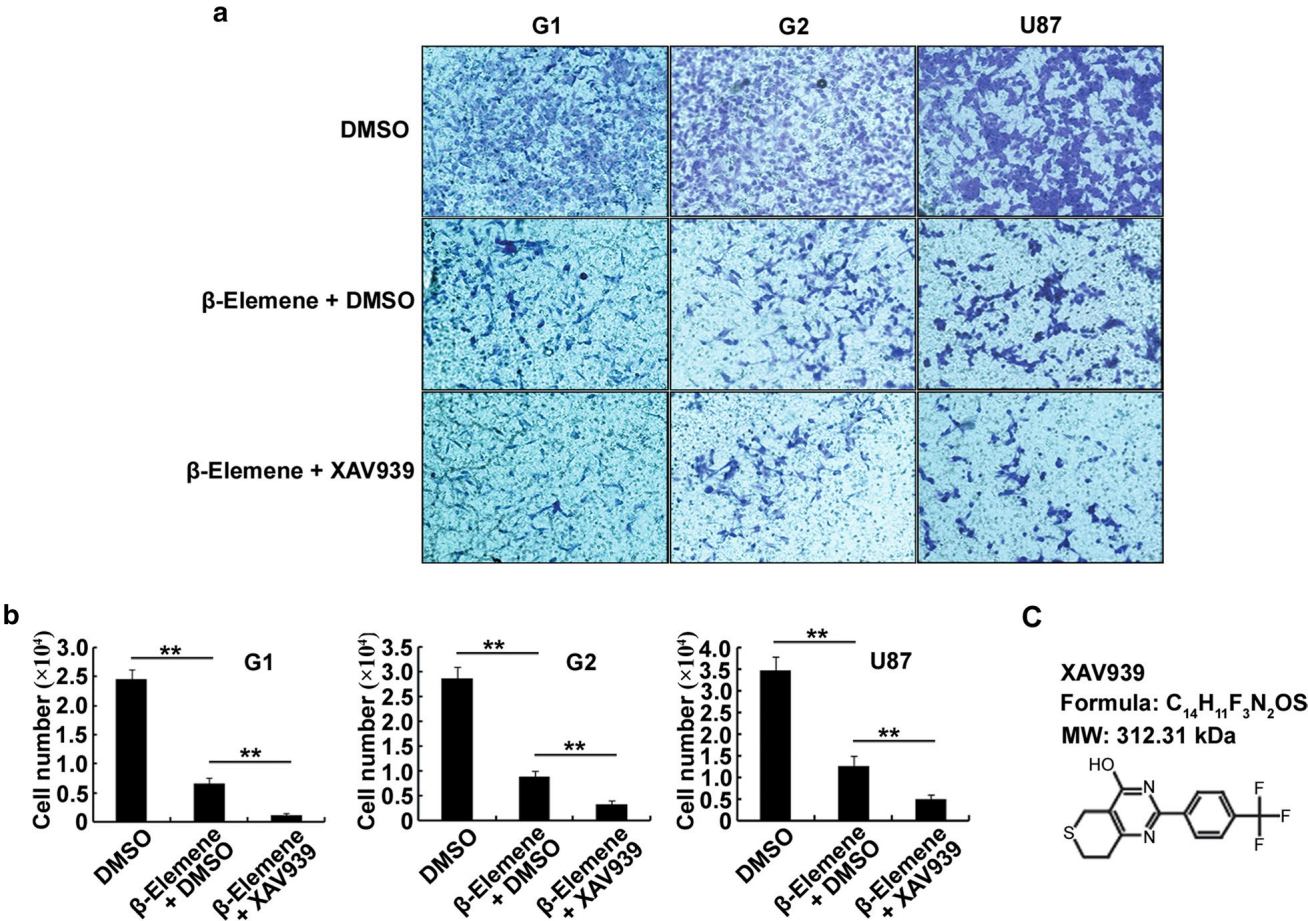
*β*-Elemene impaired the invasiveness of glioblastoma cells by reducing the expression of *β*-catenin. **a** Glioblastoma cells were separated into the following three groups: DMSO, 50 µg/ml *β*-elemene + DMSO, and 50 µg/ml *β*-elemene + 10 µM XAV939 (dissolved in DMSO) for 12 h. Transwell assays were performed to determine the invasiveness of the cells. **b** The results of (**a**) are illustrated graphically. **c** The molecular structure of XAV939. The results are representative of three independent experiments, and the values are presented as the mean ± SD (*p < 0.05, **p < 0.01)

Transwell assays were performed to further determine the effect of *β*-elemene on EMT in glioblastoma cells. To evaluate the role of the *β*-catenin signaling pathway, we also used the *β*-catenin inhibitor XAV939. Treatment conditions of 50 µg/ml *β*-elemene for 12 h were selected because this treatment did not markedly decrease the number of cells. We examined three treatment groups: DMSO, 50 µg/ml *β*-elemene + DMSO, and 50 µg/ml *β*-elemene + 10 µM XAV939 (dissolved in DMSO) for 12 h. We observed decreased invasion of G1, G2 or U87 cells in the *β*-elemene + DMSO group compared with the DMSO alone control group. Notably, the *β*-elemene + XAV939 groups showed further decreased invasion compared with *β*-elemene + DMSO group (Fig. 6). Together these results indicated that *β*-elemene decreased the invasiveness of glioblastoma cells and that this effect was amplified by the *β*-catenin inhibitor XAV939.

### Anti-glioblastoma effects of *β*-elemene were enhanced by inhibiting *β*-catenin expression

**Fig. 7:**
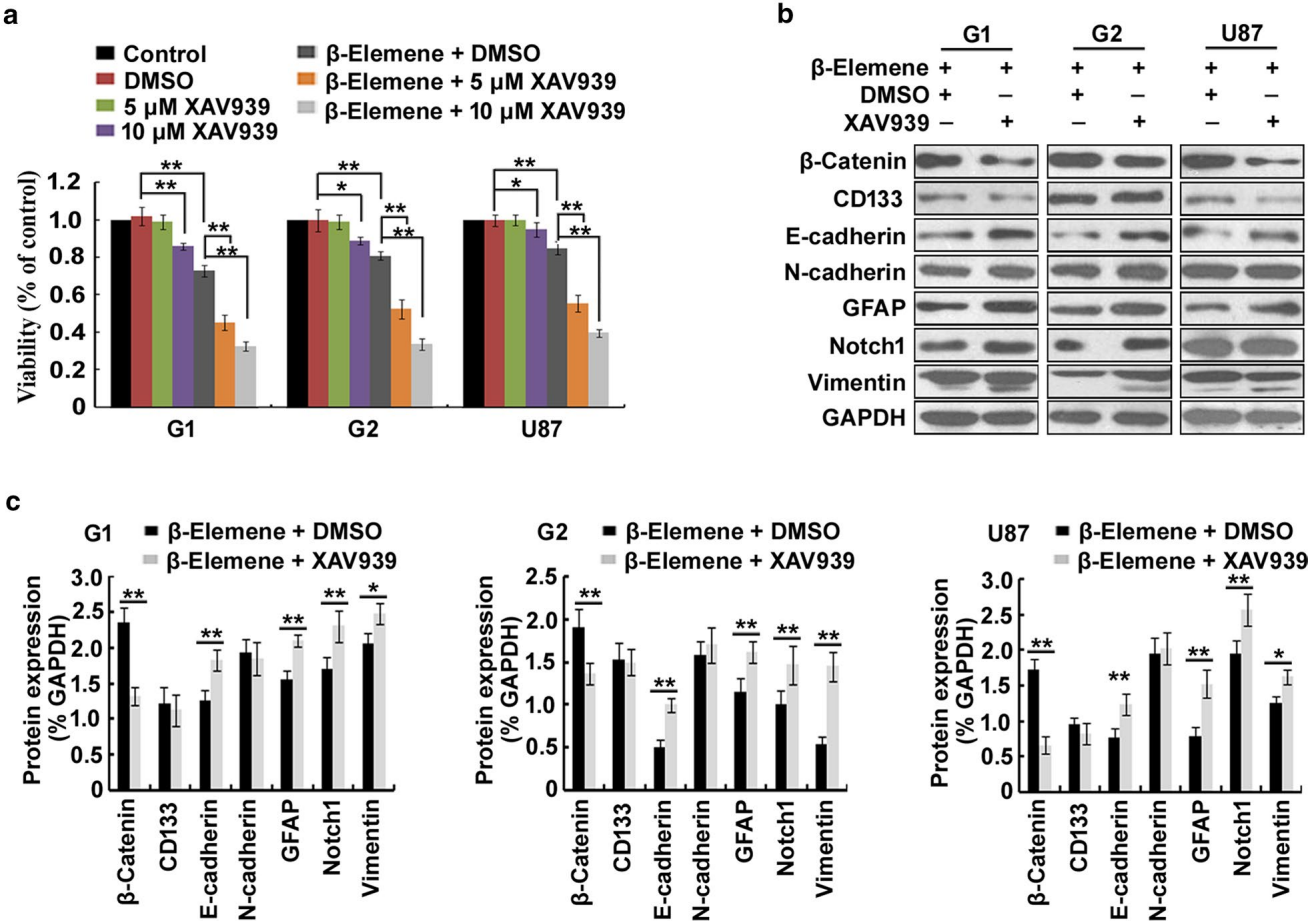
Role of *β*-catenin in the anti-glioblastoma activities of *β*-elemene. G1, G2 or U87 cells were treated with 50 µg/ml *β*-elemene in the presence or absence of XAV939 for 24 h. **a** Cell viability was determined using CCK-8 assay. Treatment with XAV939 increased the anti-proliferative effect of *β*-elemene. **b** Treatment with 10 µM XAV939 markedly downregulated the expression of *β*-catenin and upregulated the expression of E-cadherin, GFAP, Notch1 and vimentin as shown by Western blot analysis. **c** The results of (**b**) were estimated using the Gel-Pro Analyzer 4.0 software and are illustrated graphically. The results are representative of three independent experiments, and the values are presented as the mean ± SD (*p < 0.05, **p < 0.01)

To evaluate the role of the *β*-catenin signaling pathway in the anti-glioblastoma effects of *β*-elemene, we treated glioblastoma cells with 50 µg/ml *β*-elemene in the presence or absence of the *β*-catenin inhibitor XAV939 for 24 h. CCK-8 assay results revealed that treatment with 5  or 10 µM XAV939 markedly augmented the anti-proliferative effect of *β*-elemene (Fig. 7a). Western blot analysis showed that XAV939 at 10 µM clearly reduced the expression of *β*-catenin and upregulated the expression levels of E-cadherin, GFAP, Notch1 and vimentin but did not alter the expression levels of CD133 or N-cadherin (Fig. 7b, c). These results suggested that the *β*-catenin signaling pathway was involved in the anti-proliferative effect of *β*-elemene and mediated the regulation of E-cadherin, GFAP, Notch1 and vimentin expression.

### *β*-Elemene suppressed tumor development in nude mice transplanted with glioblastoma cells

**Fig. 8:**
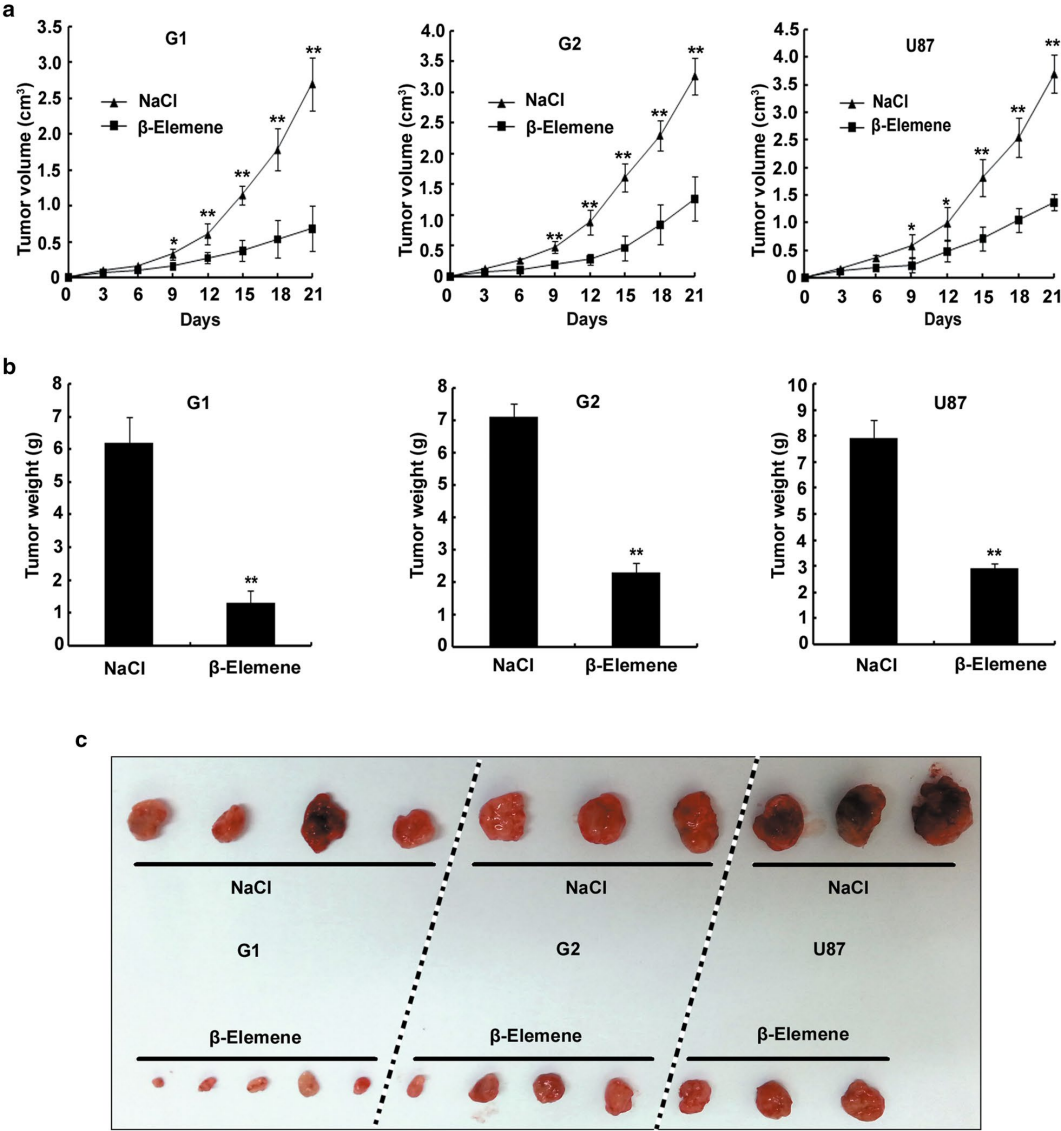
Tumor development was suppressed by *β*-elemene in nude mice transplanted with glioblastoma cells. Nude mice transplanted with glioblastoma cells received an intraperitoneal injection of NaCl or 50 mg/kg *β*-elemene for 1 week, and the tumor volumes were measured every 3 days. **a** In contrast with the NaCl-treated group, a remarkable suppression of tumor growth was detected from day 9 after transplantation (day 3 after intraperitoneal injection) to day 21 in the *β*-elemene-treated group of G1, G2 or U87 cell-transplanted nude mice. **b**, **c** The tumor weights in the *β*-elemene group on day 21 after transplantation were significantly less than those of the NaCl group. The values are presented as the mean ± SD (*p < 0.05, **p < 0.01)

To evaluate the effect of *β*-elemene on glioblastoma in vivo, we subcutaneously injected G1, G2 or U87 cells into the flank of nude mice and then intraperitoneally injected NaCl or 50 mg/kg *β*-elemene for 1 week. A total of 8 of the 30 nude mice died during days 13–20 owing to reciprocal biting. We observed a significant reduction in the tumor volume in the *β*-elemene-treated group compared with the NaCl-treated group from day 9 after transplantation (day 3 after intraperitoneal injection) to day 21 (Fig. 8a). The tumors were resected and measured on day 21. The tumor weights of the *β*-elemene-treated group were significantly less than those of the NaCl-treated group (Fig. 8b, c). These results suggested that tumor development was suppressed by *β*-elemene in tumor-bearing nude mice.

### *β*-Elemene regulation of the expression of various stemness-, differentiation- and EMT-related effectors in tumor xenografts in nude mice

**Fig. 9:**
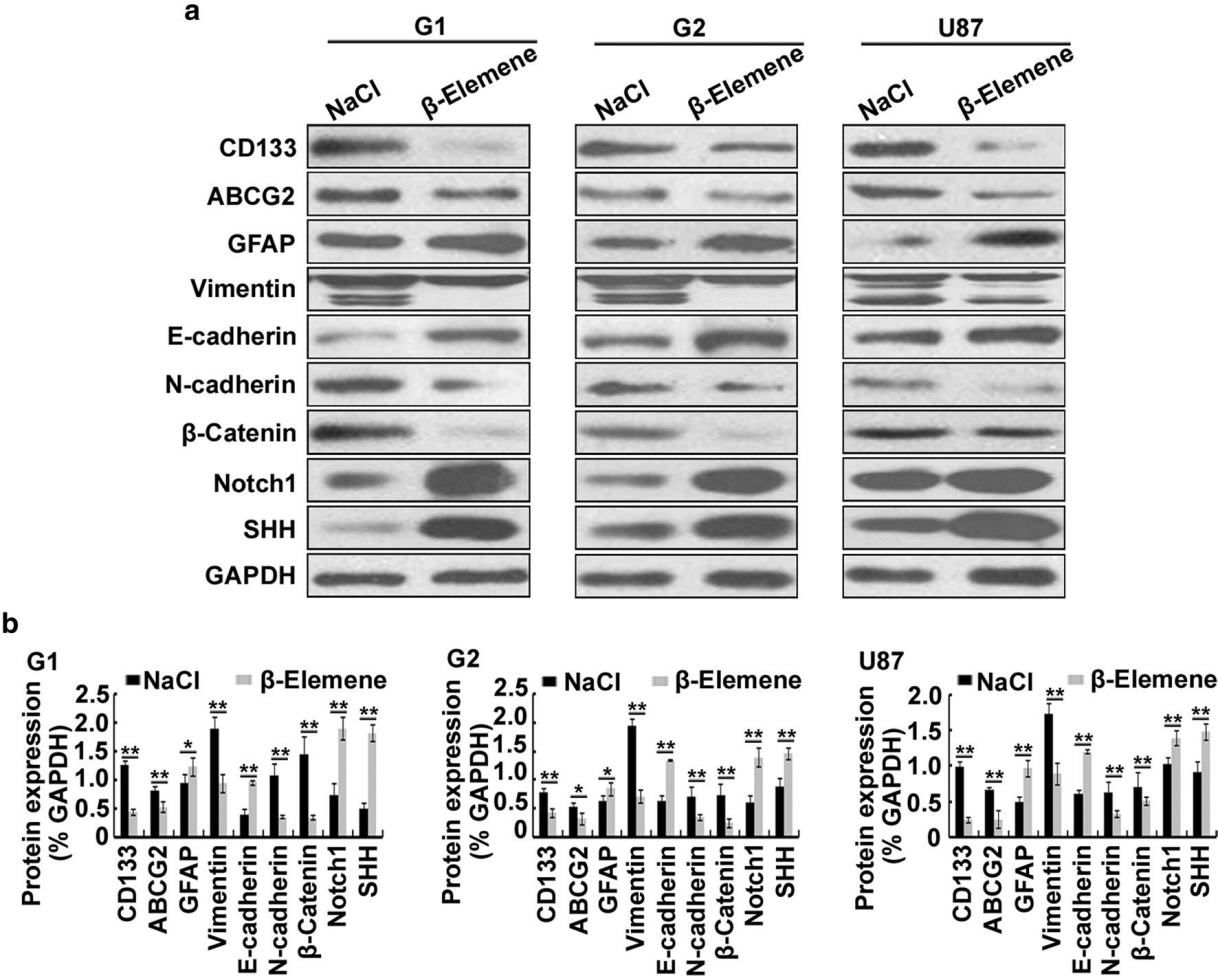
*β*-Elemene regulation of stemness-, differentiation- and EMT-related effectors in vivo. **a** Western blot assays were performed on the aforementioned glioblastoma xenografts in the *β*-elemene- and NaCl-treated groups. Expression levels of CD133, ABCG2, N-cadherin and *β*-catenin were significantly downregulated and expression levels of E-cadherin, GFAP, Notch1 and SHH were upregulated by *β*-elemene. Interestingly, the expression level of vimentin in the *β*-elemene-treated group was significantly lower than that in the NaCl-treated group; this result was opposite that for the in vitro procedure. **b** The results are representative of three independent experiments, and the values are presented as the mean ± SD (*p < 0.05, **p < 0.01)

To evaluate the effect of *β*-elemene on the expression of stemness-, differentiation- and EMT-related effectors in vivo, we evaluated the expression of CD133, ABCG2, GFAP, Notch1, SHH, vimentin, E-cadherin, N-cadherin and *β*-catenin in the aforementioned tumor tissues from glioblastoma cell-transplanted nude mice in the *β*-elemene- and NaCl-treated groups by Western blot. We found that the expression levels of CD133, ABCG2, N-cadherin and *β*-catenin were significantly lower in the *β*-elemene-treated group than those in the NaCl-treated group and that the expression levels of E-cadherin, GFAP, Notch1 and SHH were higher in the *β*-elemene-treated group than those in the NaCl-treated group of G1, G2 and U87 cell-transplanted mice (Fig. 9). Interestingly, *β*-elemene downregulated the expression of vimentin in vivo; this result was opposite that of the in vitro procedure. These results suggested that *β*-elemene regulates the expression of various stemness-, differentiation- and EMT-related effectors in vivo.

## Discussion

GSCs are considered to be an origin of tumor development, recurrence and drug resistance [30]. The radioresistance and chemoresistance of TSCs markedly decreased after their differentiation into non-stem tumor cells that express characteristic differentiation markers [31, 32]. In this study, we found that CD133^+^ and ABCG2^+^ cells were sparsely distributed throughout both the G1 and G2 glioblastoma tissues. *β*-Elemene treatment resulted in significantly decreased expression levels of CD133 and ABCG2 and increased levels of the differentiation marker GFAP in vitro and in vivo. These results suggested that *β*-elemene showed potential effects in impairing stemness and promoting differentiation in glioblastoma cells.

Notch1 and SHH play important and complex roles in the proliferation and differentiation processes of tumor cells. For instance, during the development of non-small cell lung cancer, Notch2 mediated cell differentiation, whereas Notch1 promoted the initiation and development of lung cancer [33]. Attenuating Notch1 signaling mediated the suppressive effect of enhancer-of-split and hairy-related protein 1 on EMT and metastasis in endometrial cancer [34]. However, upregulating the expression of Notch1 may inhibit proliferation and induce differentiation in many tumors. For example, the expression levels of Notch1 and GFAP were high in low-grade glioma and differentiated glioma cells but were low in high-grade glioblastoma cells and GSC spheres. Notch1 and GFAP were considered to be markers of differentiated glioma cells [21]. cAMP promotes the differentiation of rat C6 glioma cells by activating Notch1 expression [35]. Additionally, Notch1 activation also induced cellular differentiation in anaplastic thyroid cancer [36]. The inactivation of the SHH/GLI1 pathway mediated the anti-glioma effects of curcumin [37]. However, upregulating SHH expression may drive TSCs into the cell cycle and increase the susceptibility of tumor cells to chemoradiotherapy. The activation of the SHH pathway was involved in the diosgenin-induced differentiation of human erythroleukemia cells and mediated the differentiation of mesenchymal stem cells into neuron-like cells [22, 23]. In this study, we found that the expression levels of Notch1 and SHH were increased by *β*-elemene in vitro and in vivo. Upregulating the expression levels of Notch1 and SHH likely contributed to the effect exerted by *β*-elemene on glioblastoma cell differentiation.

EMT is a complex molecular and cellular program in which epithelial cells lose their differentiated characteristics, including apical-basal polarity, cell–cell adhesion, and a lack of cell motility, and gain mesenchymal properties, such as motility, invasiveness and increased resistance to apoptosis [38]. The expression of epithelial markers (such as E-cadherin) decreases and the expression of mesenchymal markers (such as vimentin and N-cadherin) increases during EMT [24, 25]. The Wnt/*β*-catenin pathway also plays a crucial role in the EMT in tumor cells. In this study, we found that *β*-elemene reduced the invasiveness of glioblastoma cells in vitro and further showed that *β*-elemene inhibited EMT by upregulating the expression levels of E-cadherin and downregulating the expression levels of N-cadherin and *β*-catenin in glioblastoma cells in vivo and in vitro.

Interestingly, the expression level of the mesenchymal marker vimentin was increased in vitro by *β*-elemene; this result was opposite as observed in vivo. We propose the following four reasons to explain this contradictory phenomenon. (1) The in vitro culture conditions cannot accurately simulate the tumor microenvironment. (2) The changes in cell morphology in vitro in response to drug treatment, including cell–cell dissociation, cell protuberance retraction, rounded morphology, and even detachment from the culture dish surface, are distinct from the morphologic changes in vivo. The expression of vimentin, an intermediate filament protein, is closely associated with alterations in cell morphology, which may partially explain the contradictory expression of vimentin in vitro and in vivo. (3) A previous study showed that increased expression of vimentin was involved in the apoptosis of Jurkat leukemia cells [39]. Thus the expression of vimentin may be affected by apoptosis in vitro. (4) A previous study showed that the number of vimentin-positive cells increased during the differentiation process of GSC spheres [40]. Therefore, cell differentiation may be typically accompanied by increased vimentin expression. The precise reason underlying the opposite effects of *β*-elemene on vimentin expression in vitro and in vivo must be validated in the future.

*β*-Catenin, a member of a protein complex that connects cadherin to the actin cytoskeleton at adherens junctions [41], plays a crucial role in tumor cell proliferation and EMT initiation and progression. Activation of the *β*-catenin/Wnt signaling cascade during EMT was associated with suppression of E-cadherin expression [42], and suppressing the *β*-catenin pathway inhibited proliferation, induced terminal differentiation and increased the expression of GFAP in glioma cells [43]. A complex crosstalk exists between the *β*-catenin pathway and various differentiation- and EMT-related effectors. Activation of *β*-catenin signaling at the cerebellar ventricular zone led to a reduction in the number of cells expressing the glial lineage markers Sox9 and GFAP [44]. Activating the Notch1 signaling pathway, which depends on GSK3*β*/*β*-catenin, inhibited cell proliferation and induced apoptosis in the human esophageal squamous cell carcinoma cell line EC9706 [45]. *β*-Catenin interacts with the intracytoplasmic region of E-cadherin to maintain cell–cell adhesion. E-cadherin and vimentin are regulated by *β*-catenin during EMT in gastric cancer [46, 47]. In this study, we found that inhibiting *β*-catenin using XAV939 enhanced the anti-proliferative and EMT inhibitory effects of *β*-elemene on glioblastoma cells and promoted the regulatory effect of *β*-elemene on EMT- and differentiation-related molecules. Our findings show that the *β*-catenin pathway plays a crucial role in the anti-glioblastoma effects of *β*-elemene. Further clarification of the regulatory mechanisms and actions of *β*-catenin signaling in anti-glioblastoma activities of *β*-elemene are required and should be pursued in future studies.

## Conclusions

*β*-Elemene clearly inhibited cell proliferation, induced cell apoptosis, promoted cell differentiation and reversed EMT by regulating the expression of a series of stemness-, differentiation- and EMT-related molecules in glioblastoma cells in vitro and in vivo. The *β*-catenin pathway played a crucial role in the anti-tumor action of *β*-elemene. This study provides promising evidence supporting *β*-elemene as a valuable future agent for glioblastoma therapy.

## Abbreviations

EMT: epithelial-to-mesenchymal transition
GSC: glioblastoma stem cell
ABCG2: ATP-binding cassette subfamily G member 2
NSC: neural stem cell
TSC: tumor stem cell
GFAP: glial fibrillary acidic protein
SHH: sonic hedgehog
CCK-8: cell counting kit-8
PI: propidium iodide
MRI: magnetic resonance imaging
DMEM: Dulbecco’s modified eagle’s medium
SD: standard deviation
